# Potential clinical applications of bimodal PET-MRI or SPECT-MRI agents†

**DOI:** 10.1002/jlcr.3154

**Published:** 2014-01-07

**Authors:** Rafael T M de Rosales

**Affiliations:** Department of Imaging Chemistry & Biology, Division of Imaging Sciences and Biomedical Engineering, King's College LondonSt. Thomas' Hospital, London, SE1 7EH, UK

**Keywords:** medical imaging, PET-MRI, SPECT-MRI, multimodal agents, SPIO

## Abstract

The introduction to the clinic of positron emission tomography-magnetic resonance imaging scanners opens up the possibility to evaluate the real potential of bimodal imaging agents. In this mini-review, the limitations in the design and applications of these materials are summarised and the unique properties that may result in real clinical applications outlined. © 2013 The Authors. Labelled *Compounds and Radiopharmaceuticals* published by John Wiley.

## Introduction

### Bimodal imaging techniques

There is an increasing interest in the combination of radionuclide-based techniques such as positron emission tomography (PET) and single photon emission computed tomography (SPECT) with non-radionuclide techniques such as computed tomography (CT) and magnetic resonance imaging (MRI). Using these combinations, imaging scanners nowadays are capable of providing molecular and physiological information with remarkable sensitivity using PET and SPECT radiotracers and, at the same time, anatomical information of the tissues of interest.

The combination of PET (or SPECT) with MRI is highly synergistic when we compare it with its predecessor PET/SPECT-CT.^1^ First, MRI does not emit ionising radiation. As a result, patients do not receive the large radiation doses from the high-energy X-rays of CT. Second, MRI has an excellent soft-tissue and temporal resolution, whereas CT is better for imaging bones and the lungs, where the presence of water (a requirement for MRI) is low. A major unique advantage of PET-MRI is the possibility of simultaneous acquisition of the two modalities; as a consequence, it is feasible to perform motion correction of the PET image for better resolution and quantification. From a clinical point of view, it is also important to mention the fact that these two important medical imaging techniques can be performed in a single appointment, a major advantage that may save time and resources to many patients and clinics. MRI is also a very versatile technique. It can image several endogenous and exogenous nuclei, mainly ^1^H from water and also others such as ^19^F, ^23^Na, or ^31^P. In addition, the signal of these nuclei is sensitive to their microenvironment, allowing the detection of these changes and to obtain spectroscopic and metabolic information from the molecules that contain them. For example, contrast agents can be developed that change (increase or decrease) the signal of water or contain exogenous nuclei (e.g., ^19^F or hyperpolarised ^13^C), allowing the detection of their location.

### Imaging agents

One of the new avenues that PET-MRI opens is the use of contrast agents in both imaging techniques. This can be carried out in two ways (Scheme 6): (a) using cocktails of imaging agents from each technique or (b) by adding a second modality component to a unimodal agent thus making it bimodal

- Cocktails of imaging agents: In this case, the idea is to obtain complementary information from both techniques. It could be quite useful to assess simultaneously two parameters about a lesion using, for example, ^18^F-FDG to obtain molecular information of the metabolism of a tissue, and at the same time, use an MRI contrast agent such as Gd-DTPA in MRI to assess the perfusion of that tissue.^2^ One could think of several PET-MR contrast agent cocktails of this kind that may result in useful biomarkers of disease.
- Bimodal agents: The second possibility, which is the focus of this mini-review, is to use contrast agents that are detectable using the two imaging modalities. The basic idea is that by having two imaging reporters, the properties of both modalities can be synergistically combined. However, the obvious limitation is the different sensitivities of the two imaging techniques. Thus, whereas PET and SPECT have very high sensitivities (i.e. nanomolar or lower concentrations of imaging agent required), MRI has very low sensitivity (i.e. millimolar concentrations required). This enormous difference makes the addition of MRI reporters to radiotracers unrealistic. However, the opposite situation (the addition of radionuclides to an MRI contrast agent) is feasible. In this case, the different concentrations necessary for detecting both imaging signals require radiolabelling with very low specific activities (radioactivity/mass of MRI contrast agent).

**scheme 1:**
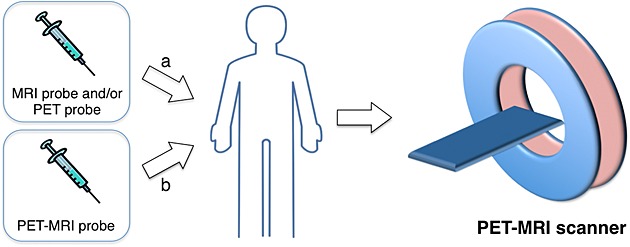
Schematic representation of the two methods to use imaging agents for positron emission tomography-magnetic resonance imaging (PET-MRI) applications.

Given these limitations, the question that remains is what could be the advantage of radiolabelling MRI contrast agents? To answer, we should look at the limitations of MRI contrast agents and how the addition of a radionuclide may provide an improvement:

1. Sensitivity, signal-to-noise ratios and full-body imaging: MRI has very low sensitivity and signal-to-noise ratios; as a consequence, it is difficult to detect areas of low contrast agent uptake. Addition of a PET/SPECT radionuclide to an MR contrast agent would allow detection of these low-uptake areas thanks to its high sensitivity and signal-to-noise ratio. In addition, technical and temporal limitations make it difficult to perform full-body MRI. The radionuclide signal of bimodal agents could be used to detect areas of low uptake or outside the MRI field of view to perform high-resolution MRI.
2. Quantification: One of the principles of molecular imaging is the ability to quantify the amount of contrast agent in a given tissue. However, accurate quantification of MRI contrast agents is very difficult. The superior quantification properties of SPECT and particularly PET would allow more accurate and sensitive measurements of the biodistribution of these agents, providing methods to correct for attenuation of the PET/SPECT signal that are developed for PET-MRI scanners in the same way that it is performed with PET/SPECT-CT scanners today.

### Potential clinical applications of bimodal PET/SPECT-MRI agents

#### Bimodal agents for measuring the pH of tissues in vivo

One of the great properties of MRI contrast agents is that they can be designed to be responsive (i.e. changes in relaxivity or ‘signal intensity’) to external factors such as pH. The clinical relevance of such probes is that pH is a potential biomarker of tumours and other tissues.^3^ One of the main limitations of these contrast agents, however, is that in order to provide accurate measurements, we need to know the exact concentration in the tissue of interest. To do this with MRI, particularly in areas of low or high uptake, is a very challenging and often inaccurate task. To overcome this problem, a solution is to add a radionuclide reporter that allows quantification and increases its detectability.

**Figure 1:**
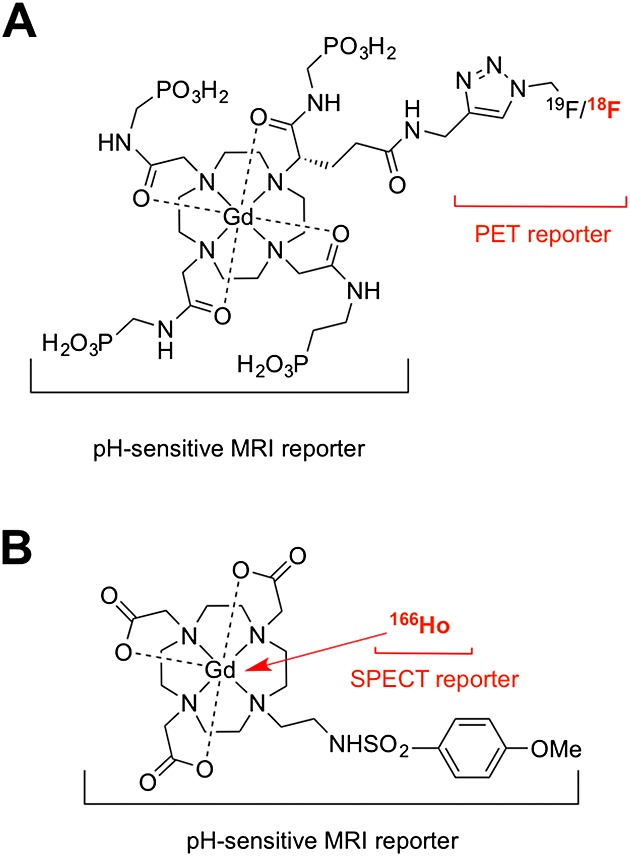
Bimodal agents for measuring the pH of tissues. (A) Gd-DOTA-4AMP-F developed by the group of Caravan *et al*.^4a^ and (B) Gd-L developed by the group of Aime *et al*.^4b^ In both cases, the positron emission tomography/single photon emission computed component is used to calculate the concentration of the contrast agent, making the pH measurement using the magnetic resonance imaging component possible.

Two fine examples of this strategy have been recently reported by Caravan *et al*. and Aime *et al*.^4^ Thus, these imaging agents (Figure 1) comprise two reporters: a gadolinium-based MRI reporter that changes its relaxivity (the ‘signal intensity’) with pH and a radionuclide that provides the sensitivity and quantification properties. This radionuclide can be added to the organic molecule by standard fluorination (^18^F) chemistry^4a^ or by exchanging some of the gadolinium atoms with a radiometal such as ^166^Ho.^4b^ As mentioned earlier, the different sensitivities of the two techniques force us to radiolabel with low specific activities, which means that both of these modifications are so small that they should not change their physicochemical properties significantly; nevertheless, it is an important factor to take into account.

#### Bimodal agents for detecting small targets

**Figure 2:**
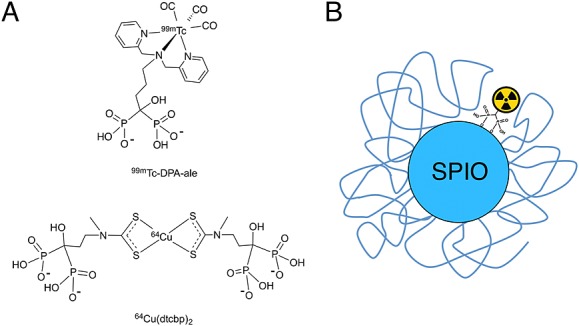
Bimodal superparamagnetic iron oxides (SPIOs) for sentinel lymph node imaging. (A) Bifunctional bisphosphonates for single photon emission computed tomography and positron emission tomography imaging used for the radiolabelling of SPIOs; (B) Schematic representation of the radiolabelled dextran-coated SPIO (Endorem/Feridex). USPIO, ultra-small SPIO.

**Figure 3:**
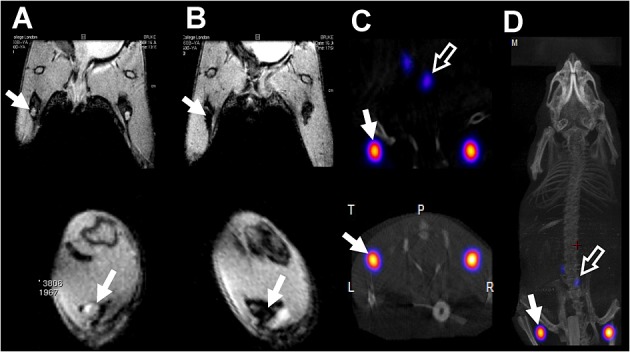
In vivo PET-MRI studies with ^64^Cu(dtcbp)_2_-Endorem in a mouse.^6b^ (A) and (B): coronal (top) and short-axis (bottom) magnetic resonance images of the lower abdominal area and upper hind legs showing the popliteal lymph nodes (solid arrows) before (A) and after (B) footpad injection of ^64^Cu(dtcbp)_2_-Endorem. (C) Coronal (top) and short-axis (bottom) NanoPET-CT images of the same mouse as in B showing the uptake of ^64^Cu(dtcbp)_2_-Endorem in the popliteal (solid arrow) and iliac lymph nodes (hollow arrow). (D) Whole-body NanoPET-CT showing sole uptake of ^64^Cu(dtcbp)_2_-Endorem in the popliteal and iliac lymph nodes. No translocation of radioactivity to other tissues was detected. PET, positron emission tomography; MRI, magnetic resonance imaging; CT, computed tomography.

**Figure 4:**
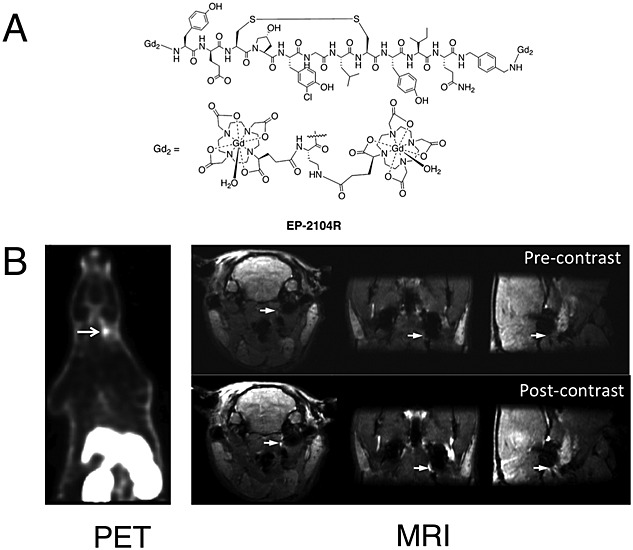
Positron emission tomography-magnetic resonance imaging (PET-MRI) of thrombi using bimodal agents.^11^ (A) EP-2104-R binds to fibrin fibres in thrombi and can be radiolabelled by partial exchange of Gd with the PET isotope ^64^Cu. (B) After injection into an animal model, ^64^Cu-EP-2104-R allows detection, localisation and quantification of the thrombi using PET-MRI with high sensitivity and spatial resolution. Arrows indicate the location of the thrombus. Image adapted with permission from the publisher of Reference 11a.

- Sentinel lymph nodes: One of the main routes of cancer spread in melanoma, breast cancer, and head and neck cancer is via the lymphatic system. To prevent this, a common clinical procedure is to surgically remove those lymph nodes that are connected to the primary tumours; these are called sentinel lymph nodes (SLNs). The most used technique to perform this today involves injecting a mixture of a radiolabelled colloid (^99m^Tc) and a blue dye into the primary tumour. This mixture slowly drains to the SLNs and allows the surgeons to use a hand-held gamma probe (sometimes aided as well by SPECT imaging) to locate them. The blue dye provides a convenient method to detect the SLNs visually during surgery in order to be able to remove them together with the primary tumour.
  This procedure has been used successfully for many years but has some drawbacks. First, these agents are nonspecific, they detect SLNs that may or may not be cancerous. As a consequence, surgeons normally remove all those SLNs they detect to prevent cancer spread. This is mostly based on the experience of each surgeon, and the outcome of the procedure may vary significantly. The second drawback is that the detection techniques are solely based on radionuclides, which give excellent detectability but have limited spatial resolution. As mentioned before, SPECT and PET have very low resolutions (in the centimetre range for human scanners), so it is possible for surgeons to miss small but cancerous SLNs. A relatively new promising method to detect SLNs with high spatial resolution is using nanoparticles based on superparamagnetic iron oxide (SPIOs).^5^ These have the advantage that MRI can detect them with high spatial resolution, allowing detection of very small SLNs and lymphatic micrometastases. A drawback of this contrast agent, however, is that it is very difficult to detect and quantify using MRI, particularly when it is confined within a very small space such as inside a lymph node or a cell. Thus, when SPIOs are in this situation, their magnetic properties change resulting in signal voids, making quantification and detection very difficult. A potential solution for this is to add a radionuclide component to SPIOs and use PET/SPECT to detect and quantify the SPIOs and also to guide high-resolution MRI of those areas. This may help to stage the disease more accurately and potentially plan the surgery.We and other groups have started work in this direction.^6^ For example, it is known that cancerous SLNs do not take up dextran-coated SPIOs but normal SLNs do.^5c^ A bimodal SPIO would allow us to detect and quantify the uptake in those SLNs (using the PET or SPECT component), avoiding the quantification problems in MRI, and also to guide high-resolution MRI to identify if they are metastatic or not. The most common method to synthesise bimodal SPIOs is to add a radionuclide-binding motif such as a chelator to the organic coating of the metallic nanoparticle.[Bibr b7][Bibr b8] Our approach consists of using small bifunctional molecules that bind radiometals such as ^99m^Tc and ^64^Cu with high in vitro and in vivo stability and that also contain a bisphosphonate group that binds very strongly to the surface of the nanoparticle (Figure 2).^6b,9^ Most labelling methods for ^64^Cu use macrocycles such as DOTA that are needed to impart the radiotracers with sufficient stability in vivo. We used a different strategy by using a dithiocarbamate group (−CS_2_) to chelate copper. The advantage of this group is that, unlike macrocycles that normally require harsh labelling conditions, it readily binds the PET isotope ^64^Cu at room temperature. In addition, it forms a *bis*-dithiocarbamate complex (^64^Cu(dtcbp)_2_, Figure 2) that contains two bisphosphonate groups that result in very strong binding to many interesting materials such as hydroxyapatite and iron oxides.^6b^ This provides a versatile method to radiolabel almost any SPIO, providing there are some exposed gaps of the metal oxide surface, which is normally the case. It should be noted, however, that the stability of Cu(II)-*bis*(dithiocarbamate) complexes is not as high as with Cu(II)-macrocycles; although when bound to the nanomaterials, the complexes have not shown any sign of decomposition in vivo. To date, we have obtained preclinical proof of concept data that shows how these bimodal SPIOs using ^64^Cu(dtcbp)_2_ Endorem can be used to detect SLNs with high sensitivity and to guide high-resolution MRI (Figure 3).^6b^
- Detection of highly abundant vascular molecular targets (e.g. fibrin in thrombi): The low sensitivity of MRI limits its use for molecular imaging. For example, to detect a single cell by MRI using a targeted agent, the number of Gd atoms must be in the order of 10^7–8^ per cell, whereas for PET/SPECT imaging, this number is around 1 radionuclide per cell.^10^ However, whereas this inherent limitation means that MR agents are unlikely to be useful for imaging low abundance targets, it still allows detection of highly abundant targets such as the components of extracellular matrix that are sometimes implicated in disease. For example, it is well known that thrombi, implicated in strokes and embolisms, contain high levels of fibrin. However, to date there is no clinical method to detect them in a whole human body. This is where PET-MRI scanners and imaging agents can be really useful. Thus, Caravan *et al*. conjugated MRI reporters based on Gd chelates to fibrin-binding peptides, and by exchanging some of the Gd with a PET radiometal (^64^Cu), a fibrin-targeted bimodal agent can be synthesised (Figure 4).^11^ These PET-MRI agents bind fibrin in vitro and in vivo with high specificity and allow the detection of thrombi anywhere in the body using the full-body capabilities of PET and guiding high-resolution MRI of its location. These probes are highly promising candidates for the detection of thrombi in humans.

**Figure 5:**
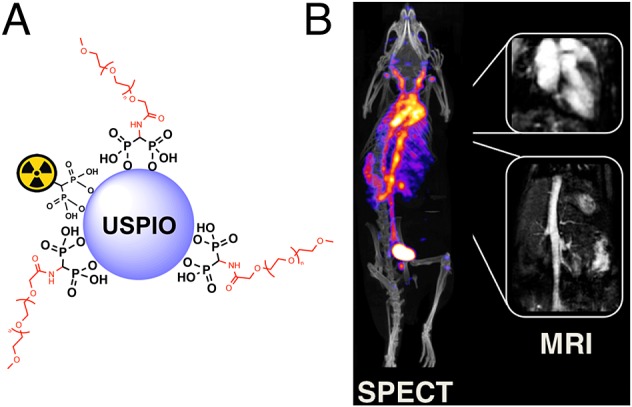
Long circulating bimodal nanoparticles for PET-MR and SPECT-MRI. (A) Bisphosphonate anchors allow strong and stable binding of PEG polymers and radionuclides on the surface of the USPIOs; (B) The bimodal nanoparticles circulate in the bloodstream, as indicated by the strong imaging signal in the heart and vessels. The compound can be detected using radionuclide imaging and T1-MRI. Negligible uptake in the reticuloendothelial system was detected.^12^ PET, positron emission tomography; MRI, magnetic resonance imaging; USPIOs, ultra-small superparamagnetic iron oxide.

Another approach to image vascular targets with PET-MRI or SPECT-MRI agents is based on nanoparticles such as SPIOs. This has the advantage that the multiplexing properties of nanoparticles allow several functionalities into the same contrast agent (targeting, stealth, multiple imaging modalities, therapy, etc.; see the succeeding text for example). One of the main problems with this approach is that, unlike for most small molecules and peptides, after injection into the bloodstream, SPIOs and nanoparticles are quickly taken by the macrophages of the reticuloendothelial system which results in short circulation times. This complicates binding to the desired target. To avoid this, we have recently prepared a new-type ultra-small SPIO with a stealth coating, based on a polyethylene glycol (PEG) polymer with bisphosphonate anchors, that efficiently prevents uptake by the reticuloendothelial system (RES) and results in bimodal SPIOs with long circulation times of 3 h (Figure 5).^12^ In addition, the bimodal agent can be detected with both radionuclide techniques (SPECT and PET) by using different bifunctional bisphosphonate tracers and T1-MRI, which is the preferred mode of obtaining images as it provides a bright, hyperintense signal similar to that of Gd compounds. We hope that this platform of bimodal SPIOs will allow us to image and quantify several vascular targets in the future with high resolution.

### Conclusions and outlook

One of the characteristics of imaging chemistry (the field of chemistry involved with the design and synthesis of imaging agents) is its interdisciplinary nature. This requires imaging chemists to interact closely with clinicians, medical physicists, imaging specialists in each of the techniques, biomedical engineers, radiochemists, and others. This interdisciplinarity is necessary to allow an understanding of what are the real clinical needs and the limitations of current techniques for the detection of disease. Bimodal imaging techniques open completely new areas of research for imaging chemists and may result in the sought-after ‘killer-application’ of PET-MRI (as ^18^F-FDG was for PET). However, the field of PET-MRI or SPECT-MRI agents is still in its infancy. One of the main barriers for researchers interested in the development of these agents is the specialised equipment and skills required to evaluate them (i.e. radiochemistry facilities, PET-MRI scanners and expertise). This is likely to change in the near future as several clinical and preclinical PET-MRI and SPECT-MRI scanners have started to be installed worldwide. It is hoped that this will result in lower economic barriers in the future and allow imaging chemists worldwide to develop and evaluate potential innovative applications of bimodal agents. Another potential barrier of this field is the higher risk of toxicity derived from the need to use MRI contrast agents—required in millimolar concentrations—although by using efficient chemistry this can be minimised.

The applications identified in this mini-review are likely candidates for clinical evaluation in the near future. Potential future applications should focus in exploiting the high sensitivity and quantification properties of PET or SPECT to detect areas of uptake and to guide high-resolution MRI. This could result in enhanced cell tracking techniques and detection of very small targets such as micrometastases. In addition, innovative applications that exploit the responsiveness of MRI agents or bimodal nature of the agents may allow the expansion of the frontiers of the field by allowing what it is not possible to do today with unimodal probes.
